# Root-associated *Streptomyces* produce galbonolides to modulate plant immunity and promote rhizosphere colonization

**DOI:** 10.1093/ismejo/wrae112

**Published:** 2024-06-19

**Authors:** Clément Nicolle, Damien Gayrard, Alba Noël, Marion Hortala, Aurélien Amiel, Sabine Grat, Aurélie Le Ru, Guillaume Marti, Jean-Luc Pernodet, Sylvie Lautru, Bernard Dumas, Thomas Rey

**Affiliations:** Laboratoire de Recherche en Sciences Végétales, Université de Toulouse, CNRS, Université Toulouse III, Toulouse INP, 24 Chemin de Borde Rouge, Auzeville, Auzeville-Tolosane 31320, France; Laboratoire de Recherche en Sciences Végétales, Université de Toulouse, CNRS, Université Toulouse III, Toulouse INP, 24 Chemin de Borde Rouge, Auzeville, Auzeville-Tolosane 31320, France; DE SANGOSSE, 47480 Pont-Du-Casse, France; Université Paris-Saclay, CEA, CNRS, Institute for Integrative Biology of the Cell (I2BC), 91198 Gif-sur-Yvette, France; Laboratoire de Recherche en Sciences Végétales, Université de Toulouse, CNRS, Université Toulouse III, Toulouse INP, 24 Chemin de Borde Rouge, Auzeville, Auzeville-Tolosane 31320, France; Laboratoire de Recherche en Sciences Végétales, Université de Toulouse, CNRS, Université Toulouse III, Toulouse INP, 24 Chemin de Borde Rouge, Auzeville, Auzeville-Tolosane 31320, France; DE SANGOSSE, 47480 Pont-Du-Casse, France; Laboratoire de Recherche en Sciences Végétales, Université de Toulouse, CNRS, Université Toulouse III, Toulouse INP, 24 Chemin de Borde Rouge, Auzeville, Auzeville-Tolosane 31320, France; Plateforme d’Imagerie FRAIB-TRI, Université de Toulouse, CNRS, Auzeville-Tolosane 31320, France; Laboratoire de Recherche en Sciences Végétales, Université de Toulouse, CNRS, Université Toulouse III, Toulouse INP, 24 Chemin de Borde Rouge, Auzeville, Auzeville-Tolosane 31320, France; Metatoul-AgromiX Platform, LRSV, Université de Toulouse, CNRS, UPS, Toulouse INP, Toulouse, France; MetaboHUB-MetaToul, National Infrastructure of Metabolomics and Fluxomics, Toulouse, France; Université Paris-Saclay, CEA, CNRS, Institute for Integrative Biology of the Cell (I2BC), 91198 Gif-sur-Yvette, France; Université Paris-Saclay, CEA, CNRS, Institute for Integrative Biology of the Cell (I2BC), 91198 Gif-sur-Yvette, France; Laboratoire de Recherche en Sciences Végétales, Université de Toulouse, CNRS, Université Toulouse III, Toulouse INP, 24 Chemin de Borde Rouge, Auzeville, Auzeville-Tolosane 31320, France; Laboratoire de Recherche en Sciences Végétales, Université de Toulouse, CNRS, Université Toulouse III, Toulouse INP, 24 Chemin de Borde Rouge, Auzeville, Auzeville-Tolosane 31320, France; DE SANGOSSE, 47480 Pont-Du-Casse, France

**Keywords:** rhizosphere, galbonolides, Streptomyces, Arabidopsis, camalexin

## Abstract

The rhizosphere, which serves as the primary interface between plant roots and the soil, constitutes an ecological niche for a huge diversity of microbial communities. Currently, there is little knowledge on the nature and the function of the different metabolites released by rhizospheric microbes to facilitate colonization of this highly competitive environment. Here, we demonstrate how the production of galbonolides, a group of polyene macrolides that inhibit plant and fungal inositol phosphorylceramide synthase (IPCS), empowers the rhizospheric *Streptomyces* strain AgN23, to thrive in the rhizosphere by triggering the plant’s defence mechanisms. Metabolomic analysis of AgN23-inoculated *Arabidopsis* roots revealed a strong induction in the production of an indole alkaloid, camalexin, which is a major phytoalexin in *Arabidopsis*. By using a plant mutant compromised in camalexin synthesis, we show that camalexin production is necessary for the successful colonization of the rhizosphere by AgN23. Conversely, hindering galbonolides biosynthesis in AgN23 knock-out mutant resulted in loss of inhibition of IPCS, a deficiency in plant defence activation, notably the production of camalexin, and a strongly reduced development of the mutant bacteria in the rhizosphere. Together, our results identified galbonolides as important metabolites mediating rhizosphere colonization by *Streptomyces*.

## Graphical Abstract

**Graphical Abstract:**
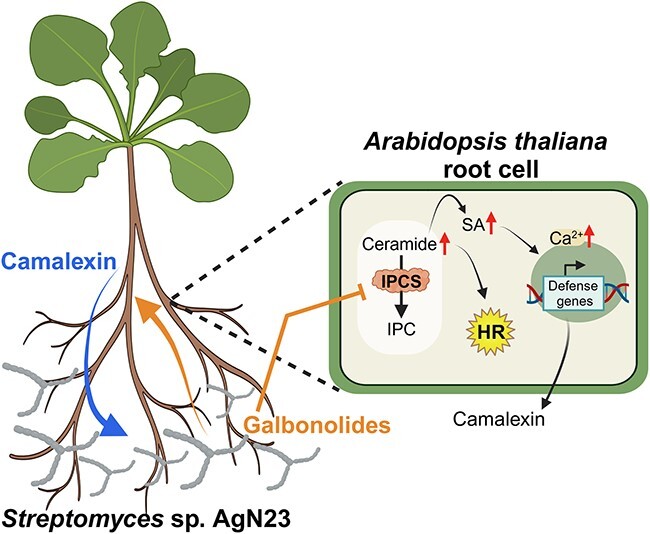

**Model summarizing the mode of action of galbonolides in stimulating plant defence to support AgN23 colonization of the rhizosphere.** Galbonolides secretion by *Streptomyces* sp. AgN23 trigger inositol phosphorylceramide synthase (IPCS) inhibition in *Arabidopsis* root cells (orange arrow). The resulting raise in Ceramide precursors of the IPCS may result in the different defence responses associated to AgN23: Hypersensitive Responses (HR), Salicylic Acid (SA) signalling, nuclear Ca^2+^ influx, defence gene expression and camalexin biosynthesis. This production of camalexin (blue arrow) exert a positive effect on AgN23 growth in the rhizosphere, presumably by restricting the growth of bacterial and fungal competitors sensitive to this phytoalexin. In addition, galbonolides secretion in the rhizosphere may also directly interfere with fungal competitors of AgN23. The illustration was created with BioRender.com.

## Introduction

Cross-kingdom communications play a significant role in shaping interactions between organisms within diverse ecological niches [1]. Microbe–microbe communication is often mediated by the secretion of small and diffusible specialized metabolites [2–6]. Throughout their lifecycle, eukaryotic organisms such as plants, are known to associate with the abundant and diverse community of microorganisms [7–9]. However, there is currently limited knowledge on how plants establish communication with microorganisms and regulate their populations in and around their tissues [10]. Plants, even when grown in geographically distant soils, tend to assemble a core microbiota comprising bacteria, fungi, and oomycetes, suggesting the existence of broad trans-kingdom communication mechanisms within plant–microbe interactions [11]. In this context, it becomes primordial to understand the molecular basis of plant–microbiota assembly to achieve the intelligent engineering of crops microbiota [12]. Such approach would be an important milestone towards sustainable agricultural practices in nutrition, protection against pathogens, and abiotic stresses [13, 14]. An example of this approach is the recently reported study on how a *Streptomyces* strain alleviates abiotic stress in a plant by producing pteridic acid [15].

The *Streptomyces* genus belongs to Actinomycetes, a family of filamentous sporulating Gram+ bacteria which constitutes the second most prominent component of root microbiota after proteobacteria [16, 17]. Among Actinomycetes, *Streptomyces* spp. are enriched in endophytic or epiphytic root compartments, and represent up to 30% of the total bacterial operational taxonomic units (OTUs) [18]. Enrichment of *Streptomyces* spp. in soil and rhizosphere correlates with resistance to drought and pathogen attack [7, 19]. Furthermore, streptomycetes are hallmark producers of antimicrobial specialized metabolites involved in protection against plant pathogens [20–22]. *Streptomyces* spp. have also been demonstrated to elicit salicylic acid (SA) and induced systemic resistance dependent responses leading to the activation of plant defence metabolism [23, 24]. These important attributes have stimulated great interest in the use of streptomycetes for crop protection [25, 26].

Previously, we reported the screening of a collection of 35 *Streptomyces* strains isolated from agricultural soils for their plant defence elicitation [27]. Among these, the AgN23 strain has been reported to display a potential to elicit *Arabidopsis* defences associated to salicylate, jasmonate, and ethylene signalling [27]. Foliar inoculation with the bacteria resulted in the formation of *SALICYLIC INDUCTION DEFICIENT 2* (*SID2*) dependent necrotic symptoms in *Arabidopsis* and protection against *Alternaria brassicicola* colonization [27]. A recent analysis of the AgN23 genome showed that the strain belongs to the clade *Streptomyces violaceusniger* [28]*.* The AgN23 genome harbours large gene families associated to rhizosphere colonization, such as biosynthetic gene clusters (BGCs) involved in the synthesis of plant bioactive and antimicrobial compounds, plant cell wall degrading enzymes, and phytohormone synthesis.

In this study, we investigate the molecular basis of AgN23 interaction with plant roots by characterizing rhizosphere colonization by the bacteria and the resulting plant responses. We find that AgN23 triggered plant biosynthesis of the antimicrobial camalexin and show that this phytoalexin is an important feature for rhizosphere colonization by the *Streptomyces*. In addition, we established that AgN23 produce galbonolides that can interfere with plant sphingolipid metabolism by targeting the inositol phosphorylceramide synthase (IPCS). Finally, we show that galbonolides biosynthesis by AgN23 is instrumental for plant defence stimulation, including camalexin production and rhizosphere colonization by the bacterium.

## Material and methods

### Plant material, growth conditions and phenotyping

Seeds of *Arabidopsis thaliana* accession Col-0 (N1092) were obtained from the Nottingham Arabidopsis Stock Centre and mutant *pad3–1* (N3805) were kindly provided by Dr. Pawel Bednarek. *Arabidopsis* plants grown in potting soil (PROVEEN; Bas Van Buuren B.V., Holland) were cultivated in a growth chamber under 16 hours photoperiod and 23°C unless otherwise indicated. Similar conditions were applied to the cultivation of *Nicotiana benthamiana*. One-month-old *N. benthamiana* leaves were syringe-infiltrated with bacterial culture media extracts (CMEs). Cell death areas were photographed 24–48 hours after infiltration, with an Expression 11000 XL scanner (Epson) at 300 dots/inch.

To perform soil inoculation assays with AgN23, 70 g of potting soil inoculated with AgN23 spore inoculum at 10^4^ CFU/g was distributed in pots placed in small plastic bags to avoid cross-contamination during watering. About 5 to 10 *Arabidopsis* seeds were sown per pot and the pots were placed in a growth phytotronic chamber. A single seedling was kept per pot 5 days after germination. Pots were watered weekly with 10 ml of tap water. The watering pots were photographed to monitor the aerial part phenotype. Green area was measured with ImageJ (v. 1.51 k) at 4, 6, or 7 weeks after inoculation. Details regarding in vitro cultivation of *Arabidopsis* are in Supplementary methods.

### AgN23 cultivation and transgenesis to obtain reporter lines and Δ*gbn*B mutants

AgN23 was grown in Bennett medium for the purpose of liquid state cultivation and of CME production (d-Glucose 10 g/L; Soybean peptones 2.5 g/L; Yeast Extract 1.5 g/L; Sigma). The culture was set in 250-ml Erlenmeyer flasks by inoculating 50 ml Bennett medium with 100 μl of fresh spore suspension at 10^5^ CFU/ml at 250 rpm and 28°C in a shaking incubator at 250 rpm for 7 days. For the purpose of spore production and genetic manipulations AgN23 strain was cultivated on the solid medium Soya Flour Mannitol medium (d-Mannitol (Sigma) 20 g/L; organic soya flour (Priméal) 20 g/L; Bacto Agar (Difco Laboratories) 20 g/L).

*Escherichia coli* strains were grown in LB with appropriate antibiotics as necessary. *E. coli* transformation and *E. coli* / *Streptomyces* conjugation were performed according to standard procedures [29]. Phusion High-fidelity DNA Polymerase (Thermo Fisher Scientific) was used to amplify DNA fragment except for PCR verification of plasmids or strains for which Taq polymerase (Qiagen) was used. DNA fragments and PCR products were purified using the Nucleospin Gel and PCR clean up kit (Macherey-Nagel).

For pOSV700 plasmid construction, a 0.4 kb DNA fragment encompassing the ermEp^*^ promoter and the tipA ribosome binding site was amplified from pOSV666 using the primers JWseq6 and JWseq7. The fragment was digested by EcoRV and cloned into EcoRV-digested pSET152, resulting in pOSV700. The sequence of the insert was verified.

For GFP and mCherry transgenesis, the sequences of the soluble-modified GFP (smGFP) and mCHERRY genes were optimized for expression in *Streptomyces*, synthesized as gblocks (IDT) and cloned into pGEM-T easy, resulting in pmsolGFP and pmCHERRY, respectively. The smGFP and mCHERRY genes were amplified from these plasmids using the primer pairs onSC001/onSC011 and onSC005/onSC013, respectively. PCR amplicons were digested by NdeI and PacI and cloned into NdeI/PacI-digested pOSV700. The resulting plasmids were verified by restriction digestion, sequencing, and named pSC001 (smGFP) or pSC003 (mCHERRY). These were subsequently introduced in *E. coli* ET12567/pUZ8002 and transferred into *Streptomyces* sp. AgN23 by intergeneric conjugation. Conjugants were selected on apramycin 50 μg/ml. The resulting strains were verified by PCR on the extracted genomic DNA using the pSET152-F and pSET152-R primers.

For production of galbonolides knock-out mutants, a 5 kb internal fragment of *gbnB* coding for the structural PKS gene of the galbonolides biosynthetic gene cluster was replaced by a kanamycin resistance cassette. For this purpose, a 2 kb fragment (upstream fragment) encompassing the beginning of *gbnB* was amplified by PCR with the onSC007/onSC008 primer pair and cloned into pGEM-T Easy, yielding pSC008. Similarly, a 2 kb fragment (downstream fragment) encompassing the end of *gbnB* was amplified by PCR with the onSC009/onSC010 primer pair and cloned into pGEM-T Easy, yielding pSC009. The pSC008 and pSC009 plasmids were digested by EcoRI/EcoRV and DraI/HindIII, respectively, and the 2 kb fragments (upstream and downstream fragments respectively) were purified on agarose gel. The kanamycin resistance cassette was obtained by digesting pOSV514 by EcoRV. The three fragments (upstream, downstream and resistance cassette) were next ligated into EcoRI/HindIII-digested pOJ260. The resulting plasmid, named pSC004, was verified by digestion with BamHI, PstI, EcoRI, and EcoRV. Five independent conjugants were verified by PCR using the onSC022/onSC023, onSC021/JWseq16, and onSC030/JWseq17 primer pairs. All oligonucleotides used in this work are listed in Supplementary Table S1.

### Analysis of Arabidopsis defence response

Detailed procedures for *Arabidopsis* loss of electrolytes and Calcium signal detection are described in Supplementary Methods. For defence gene expression assays, total RNAs were extracted using the RNeasy Plant Mini Kit (Qiagen) and DNase treated with RQ1 RNase-Free DNase (Promega). For each sample, 1 μg of total RNA was reverse-transcribed with the High Capacity cDNA Reverse Transcription Kit (Applied Biosystems). cDNAs were diluted to 1 ng/μl and used for qPCR analysis in a 10 μl reaction mix containing 5 μl of LightCycler 480 SYBR Green I Master mix (Roche), 300 nM of each primer, and 2 μl of the diluted template cDNAs. qPCR was performed in triplicate using a LightCycler® 480 System (Roche) with preheating at 95°C for 5 minutes, then 40 cycles of 95°C for 15 s, and 60°C for 60 s. The Polyubiquitin 10 gene AT4G05320 was retained for normalization. The 2^-ΔCp^ method was used to display gene expression levels. Primers used in this study are listed in Supplementary Table S1.

### AgN23 DNA quantification from soil and rhizosphere DNA

To track the development of AgN23, the plants were removed from the soil. The remaining soil from each pot was homogenized, then a small amount was sampled, and considered as bulk sample. Roots were placed into 50 ml conical sterile polypropylene centrifuge tubes filled with 20 ml 1× phosphate-buffered saline (pH 7.4), and vigorously vortexed to release the adhering rhizospheric soil. Tubes were then centrifuged at 4000 rpm and the washing step was repeated one time. Soil pellets after second centrifugation step were considered as rhizosphere samples. Samples were stored at −80°C until processing. The total microbe DNA from 100 mg of bulk or rhizosphere samples was extracted using the *Quick*-DNA Fecal/Soil Microbe Miniprep kit (Zymo Research) following manufacturer’s instructions. DNA was eluted in 100 μl DNA Elution Buffer, quantified with DS-11 Spectrophotometer/Fluorometer (DeNovix), and stored at −80°C until processing. The experimental procedures and calculation for AgN23 genome copies quantification in the rhizosphere is detailed in Supplementary methods.

### Preparation of samples for biochemistry studies and mass spectrometry analysis

For root metabolome studies, 10 *Arabidopsis* seedlings from the same MS plate were sampled together in 2 ml microtubes containing two 3 mm-diameter tungsten carbide beads (Qiagen), and flash frozen in liquid nitrogen. For studies of AgN23 CME, the bacterial biomass grown in liquid flask culture was removed from the culture supernatant by centrifugation at 4200 rpm for 10 min, completely dried in oven at 50°C, and weighed to assess AgN23 growth. Further details regarding metabolites extraction of AgN23 and *Arabidopsis* are detailed in Supplementary methods.

### Microscopy

For stereo microscopy, we used a Nikon SMZ16 microscope equipped with a camera. Confocal microscopy was performed on a TCS SP8 confocal microscope (Leica, Microsystems, UK). For GFP-tagged AgN23 cells, the excitation wavelength was 488 nm, with emission absorbance between 500 and 550 nm, whereas an excitation wavelength of 543 nm was used for mCherry-tagged AgN23 cells proteins, with emission absorbance between 560 and 600 nm. Images were acquired with a × 40 or ×20 water immersion lens. All confocal images were analysed and processed using the ImageJ software package (http://rsb.info.nih.gov/ij/; v. 1.51 k).

### *Arabidopsis* inositol phosphorylceramide synthase inhibition assay

To study the effect of AgN23 CME on *Arabidopsis* IPCS, we purified microsomal fractions of transgenic yeast expressing AtIPCS2 (AT2G37940) in enzymatic activity assays. First, a preculture of yeast MSY23-3C pESC-LEU_AtIPCS2 strain was performed by picking a single colony and propagating it in 5 ml SGR -TRP -LEU medium (0.1% galactose, 1% raffinose) [30]. The preculture was incubated at 30°C, 200 rpm until the OD_600_ reached 0.8. The preculture was then mixed with 245 ml of fresh SGR medium and incubated at 30°C, 200 rpm until the OD_600_ reached 0.8. Yeast cells were then harvested by centrifugation, washed with cold phosphate-buffered saline, and stored at −80°C until microsome preparation. Crude microsomal membranes from yeast MSY23-3C pESC-LEU_AtIPCS2 strain were prepared as previously described with additional CHAPSO washing steps [31]. Total protein quantification was performed by Bradford assay and aliquots at 0.5 mg/ml were made, and stocked at −80°C. Details regarding analytical parameters to study AtIPCS2 enzymatic activity are described in Supplementary methods.

## Results

### AgN23 colonizes *Arabidopsis* rhizodermis and rhizosphere

**Figure 1:**
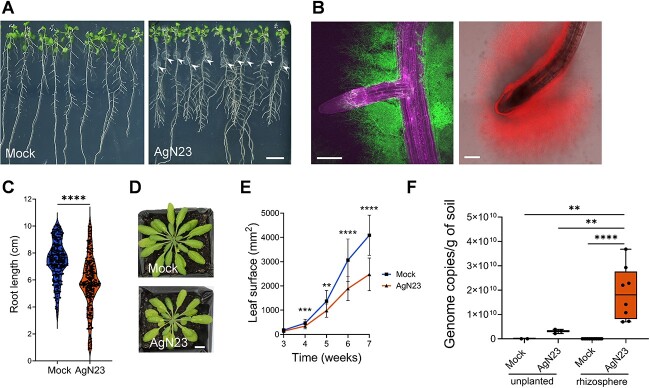
**AgN23 colonizes rhizoplane and rhizosphere of *A. thaliana* and slightly inhibits plant growth. A.** Observation of *A. thaliana* Col-0 colonization by AgN23 at 10 days after inoculation with spores at the root apex. arrowheads indicate AgN23 initial spore inoculation, scale bar = 1 cm. **B.** Confocal fluorescence images of AgN23-mCherry colonizing root apical meristem and AgN23-GFP developing around lateral root. Scale bars: 250 μm **C.** Primary root length of *Arabidopsis* seedlings 10 days after inoculation with AgN23 spores at the root apex from violin plots created from data from 30 independent assays each involving at least 10 plants per treatment (n = 300). The whiskers encompass the minimum and maximum values, and the midline shows the median. Statistical differences between the treatments were analysed using Mann–Whitney test and “^*^^*^^*^^*^” represents significant differences at *P* value <0.0001. **D.** Typical photographs of 6-week- old Arabidopsis rosettes following growth within non- or inoculated potting soil. scale bar: 1 cm. **E.** Leaf area measurement of *Arabidopsis* rosettes grown in AgN23-inoculated potting soil. graphs show the mean ± SD calculated from at least eight biological replicates (n = 8). Statistical comparison between inoculation and mock conditions was performed based on t-test (“^*^^*^” = *P* value <0.01; “^*^^*^^*^” = *P* value <0.001; “^*^^*^^*^^*^” = *P* value <0.0001). **F.** AgN23 genome copy number in *Arabidopsis* rhizosphere 4 and 8 weeks after soil inoculation. Box plots were created from data involving at least 8 plants per treatment (n = 8). Whiskers encompass the minimum and maximum values, and the midline shows the median. Statistical differences between the treatments were analysed using Mann–Whitney test; “^*^^*^^*^^*^” and “^*^^*^” represent significant differences at *P* value <0.0001 and *P* value <0.05, respectively.

In view of the fact that AgN23 was isolated from grapevine rhizosphere, we looked into the interaction of the strain with roots by inoculating in vitro grown *A. thaliana* Col-0 seedlings with AgN23 spores. A drop of the spore suspension was deposited at the root tip of young seedlings. A strong development of bacterial microcolonies was observed at the inoculation site 10 days after inoculation (Fig. 1A). We generated GFP and RFP-labelled transgenic AgN23 strains, and observed the colonization patterns of both strains by epifluorescence microscopy. Results showed that bacteria can spread beyond the initial inoculation spot and colonize other developing sections of the root system, such as lateral roots, and apical meristem (Fig. 1B). Visual and microscopic inspection of the AgN23-treated plants suggested that the inoculated bacteria did not lead to any characteristic symptoms such as root browning or rhizodermis damages in *Arabidopsis*. Moreover, penetration of AgN23 into the root tissues was not observed. Nevertheless, we observed that AgN23 inoculation did result in a slight (~10%) reduction in root elongation (Fig. 1C).

To study the colonization of the rhizosphere by AgN23, we inoculated potting soil with 10^5^ AgN23 spores/g of soil prior to sowing Arabidopsis seeds. Consistent with our previous in vitro observation, the presence of AgN23 reduced rosette growth without causing obvious symptoms to the leaves (Fig. 1D and E) [27]. The development of AgN23 in the inoculated soil was monitored by extracting microbial DNA from both unplanted and rhizosphere soil samples. A quantification of AgN23 genome copies was implemented by amplifying a specific genomic region of the strain from soil samples and from a standard curve of AgN23 purified DNA. Knowing the molecular weight of AgN23 genome, we extrapolated genome copies number from the estimated mass of AgN23 DNA detected in soil. A total of 3.06×10^8^ genome copies of AgN23 were detected in the unplanted soil 7 weeks post inoculation, whereas 1.87×10^10^ genome copies of the bacteria were detected in the rhizosphere. This shows that AgN23 preferentially colonized the *Arabidopsis* rhizosphere rather than the unplanted soil (Fig. 1F). Taken together, our data confirm that AgN23 is an epiphytic and rhizospheric bacterium that triggers slight reduction in plant growth, albeit without symptoms.

### Activation of camalexin biosynthesis by AgN23 promotes bacteria settlement in the rhizosphere

In a previous study, we characterized the plant defence stimulating activity of AgN23 and found that the bacterial CME-induced robust transcriptional responses associated with *Arabidopsis* specialized metabolism [27]. Detailed analysis showed transcriptional induction of genes coding enzymes involved in camalexin biosynthesis following treatment with AgN23 CME after 1 and 6 hours post-treatment which is a major phytoalexin of *Arabidopsis* belonging to indole alkaloid (See online supplementary material for a colour version of Supplementary Fig. S1).

To analyse the metabolomic response of root tissues to AgN23, we extracted the metabolites from whole seedlings cultivated in vitro in contact with AgN23 for 10 days. The extracts were subjected to a full-scan LC-HRMS metabolomics analysis in ESI+ and ESI- modes, and then combined in a single list of variables. A total of 511 variables were retrieved across all the samples out of which, 416 received level 3 annotations according the Metabolomic Standard Initiative based on internally built database, the exact mass, and fragmentation profile of the ions (Supplementary Table S2). Unsupervised PCA of the complete variable dataset allowed us to clearly discriminate the control samples from those inoculated with AgN23, with component 1 and component 2 supporting 32.9% and 21.6% of the variability, respectively (See online supplementary material for a colour version of Supplementary Fig. S2).

**Figure 2:**
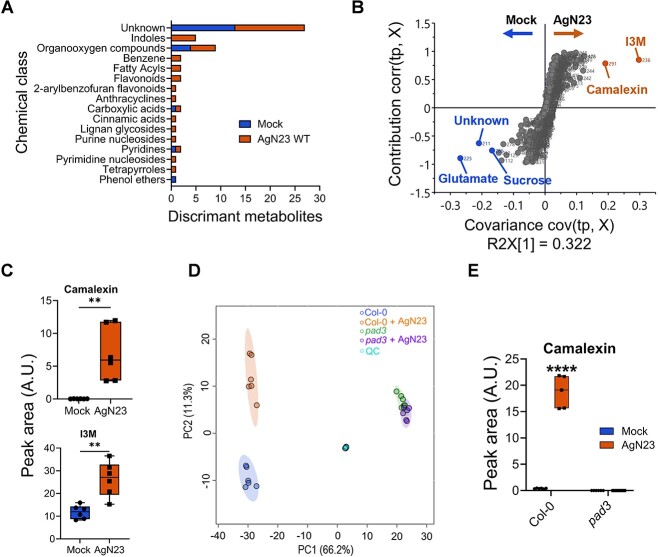
**AgN23 induces camalexin biosynthesis in *Arabidopsis* roots. A.** Discriminant metabolites overrepresented in mock or AgN23 treated *Arabidopsis* roots based on UHPLC–MS profiling data. The metabolites are displayed as chemical classes, determined with ClassyFire, using the criteria of *P* value <0.05 (t-test, control vs treatment, unadjusted *P* value) and log2 fold change (log2FC) > 0.8 or < −0.8. **B.** Corresponding S-plot of OPLS-DA score plot based on mock vs AgN23 comparison (n = 511 variables, the OPLS-DA model was validated by a permutation test with 200 counts). The variables with VIP > 3.5 are indicated for AgN23 group and mock group, respectively. **C.** Average peak area of the 2 biomarkers significantly induced in AgN23 treated roots (VIP > 3.5). Box plots were created from data from six independent assays (n = 6). The whiskers encompass the minimum and maximum values, and the midline shows the median. Statistical differences between the treatments were analysed using unpaired t-test and “^*^^*^” represents significant differences at *P* value <0.01. I3M: Indole-3-yl-methyl. **D.** PCA score plot of UHPLC–MS data (n = 534 variables) from extracts of *Arabidopsis* Col-0 or *pad3–1* 10 days after inoculation with AgN23. **E.** Average peak area of camalexin. Box plots were created from data from six independent assays (n = 6). The whiskers encompass the minimum and maximum values, and the midline shows the median.

To identify the underlying chemical classes supporting the separation of control and AgN23 inoculated samples, we computed the fold change for each individual variable between the two conditions. Results showed that 20 and 39 metabolites were enriched in control and AgN23 conditions, respectively (Supplementary Table S2). These metabolites were sorted based on their chemical classes, revealing a strong induction of metabolites belonging to specialized metabolism, such as indoles, flavonoids, or fatty acyls (Fig. 2A). A PLS-DA model was then built to identify the most significant metabolites supporting samples separation (Fig. 2B). It turned out that primary metabolism markers (sucrose and glutamate) were enriched in the control root, suggesting that these are depleted from the roots in presence of the bacteria. In contrast, camalexin, and indol-3-yl-methylglucosinolate (I3M) were the two most significant enriched metabolites in AgN23 treated roots. This suggests that the biosynthesis of these two metabolites is induced by the bacteria. This conclusion was further substantiated by comparing the peak areas corresponding to the two metabolites in mock and AgN23-treated plants. In the presence of AgN23, 259.4 and 2.2-fold induction were observed for camalexin and I3M, respectively (Fig. 2C).

In view of the strong and specific production of camalexin in response to AgN23, we characterized the behaviour of the *phytoalexin deficient mutant 3 (pad3-1),* mutated in a CYP450 coding gene which converts cysteine-indole-3-acetonitrile to camalexin, in response to the bacteria [32]. Metabolomics characterization of *pad3-1* roots indicated that the metabolome of *pad3-1* upon AgN23 inoculation was indistinguishable from that under mock conditions (Fig. 2D, Supplementary Table S3). We further validated the complete lack of induction of camalexin biosynthesis in *pad3–1* (Fig. 2E).

**Figure 3:**
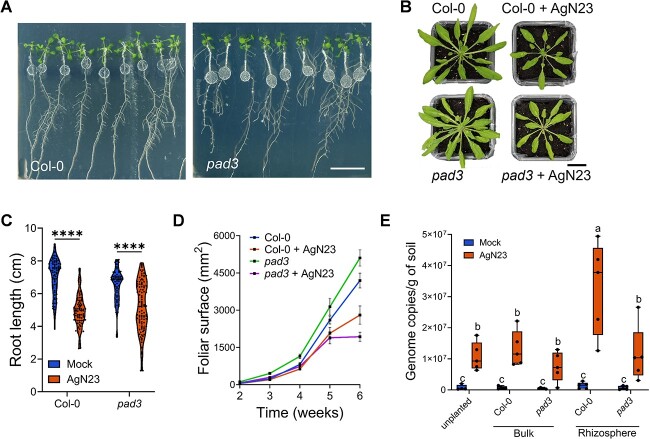
**Biosynthesis of camalexin enables enrichment of AgN23 in the *Arabidopsis* rhizosphere. A.** Observation of *A. thaliana* Col-0 and *pad3–1* colonization by AgN23 at 10 days after inoculation with spores at the root apex. scale bar = 2 cm **B.** Rosette development of the plants Col-0 and *pad3–1* after inoculation with AgN23 spores. Typical photographs of 6-week-old col-0 or *pad3–1* rosettes are shown (bar = 2 cm). **C.** Primary root length of plants colonized or not by AgN23 at 10 days after inoculation. **D.** Leaf area measurement. Graphs show the mean ± SD calculated from at least eight biological replicates (n = 8). **E.** AgN23 genome copy number in Col-0 or *pad3–1 Arabidopsis* rhizosphere 6 weeks after soil inoculation with AgN23. Box plots were created from data from 5 plants per treatment (n = 5). The whiskers encompass the minimum and maximum values, and the midline shows the median. Letters a to c represent statistical differences between the treatments based on 2-way ANOVA followed by Tukey’s multiple comparisons test.

We observed that *pad3-1* plants inoculated with AgN23 showed a phenotype similar to that of the wild type Col-0 (Fig. 3A and B) with respect to roots and rosette growth inhibitions (Fig. 3C and D). To check if camalexin production had any effect on AgN23 multiplication in the rhizosphere, we quantified AgN23 in the rhizosphere of the WT and the *pad3-1* mutant. Results showed that the number of genome copies of AgN23 in the rhizosphere of *pad3-1* plant was 2.96 times lower than in Col-0 (Fig. 3E). Taken together with the data from in vitro inoculation, these results demonstrate that the induction of camalexin synthesis promotes AgN23 colonization in the rhizosphere.

### AgN23 produces galbonolides, polyketides capable of inhibiting plant inositol phosphorylceramide synthase

To identify AgN23 specialized metabolites that could be involved in elicitation of root metabolome responses, we performed a liquid chromatography high-resolution mass spectrometry (LC-HRMS) global metabolomics analysis. Briefly, apolar compounds of the CME were adsorbed on XAD16 resin beads and extracted with butanol prior to preparation for full-scan LC-HRMS. The samples were injected in ESI+ and ESI− mode, and combined in a single list of variables. A total of 1022 variables were retrieved across all the samples and 812 received level 3 annotations according the Metabolomic Standard Initiative based on internally built database, the exact mass, and fragmentation profile of the ions (Supplementary Table S4). This approach led to the putative identification of several specialized metabolites that have been known to be produced by *Streptomyces* ssp. (See online supplementary material for a colour version of Supplementary Fig. S3 and Table 1).

**Table 1:** List of detected metabolites with highest intensities on chromatogram.

Peak name	Ret. Time (min)	m/z
Galbonolide E	7.63	365.19
Galbonolide A	7.92	379.21
Galbonolide G	8.21	363.21
Niphimycin	8.53	1140.71
Niphimycin	9.15	1140.71
Nigericin	12.41	723.47
Nigericin	15.12	723.47

**Figure 4:**
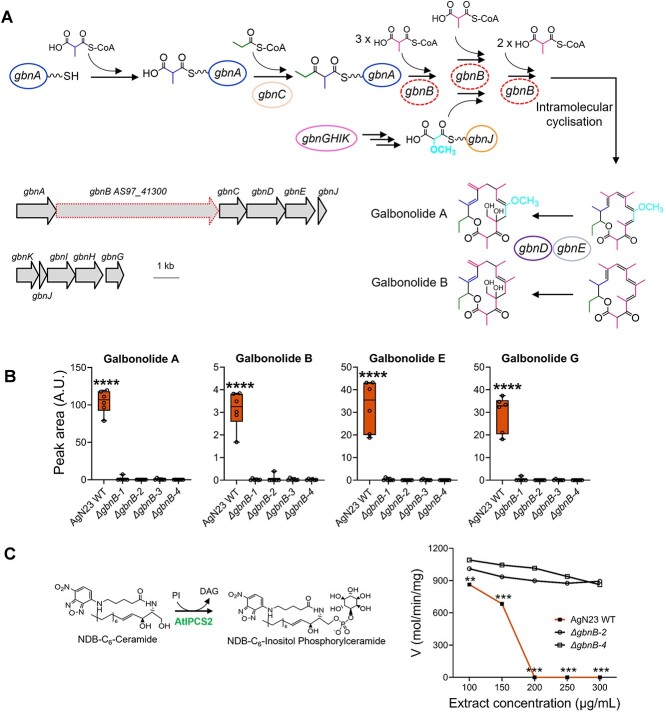
**AgN23 produces galbonolides, a class of macrolides capable of inhibiting plant inositolphosphoryl ceramide synthase. A.** Biosynthesis pathway of galbonolides (*gbn*A-E) and of methoxymalonyl-CoA (*gbn*H-K) in AgN23. The targeted locus of the AgN23 galbonolide BCG for knock-out is shown by the broken line (NCBI locus tags from assembly GCA_001598115.2). **B.** Average peak area of the different putative galbonolides structures detected in AgN23 CME based on HRMS and MS/MS spectra. Box plots were created from data from 6 biological replicates (n = 6). The whiskers encompass the minimum and maximum values, and the midline shows the median. Statistical differences between the AgN23 wild-type (WT) group and the AgN23 KO (Δ*gbnB*) groups were analysed using one-way analysis of variance (ANOVA) and Tukey’s HSD test (α = 0.05) and “^*^^*^^*^^*^” represents significant differences at *P* value <0.0001. **C.** Pathway of NBD-C6-ceramide to NBD-C6-IPC conversion by the *Arabidopsis* inositol phosphorylceramide synthase (AtIPCS2) and enzyme activity following treatments with butanol extracts from culture supernatant of AgN23 WT or KO mutants (Δ*gbnB-*2 and Δ*gbnB-4*). Graphs show the mean ± SD calculated from 6 independent assays (n = 6). Statistical differences between the AgN23 wild-type (WT) group and the AgN23 KO (Δ*gbnB*) groups were analysed using multiple Mann–Whitney test (FDR = 1%) and “^*^^*^^*^” and “^*^^*^” represent *P* value <0.001 and *P* value <0.01, respectively.

Among these specialized metabolites, we identified the antifungal compounds niphimycin, nigericin, and galbonolides (also known as rustmicin). The identification of these specialized candidate metabolites is consistent with the BGCs that we recently annotated [28]. Among the three compounds, galbonolides were originally reported for their inhibitory activities against fungal and in plants inositol phosphorylceramide synthase (IPCS), an enzyme involved in the metabolism of sphingolipids [33]. The loss of function of an IPCS gene in *Arabidopsis* has been shown to be associated with programmed cell death linked to defence mechanisms [34–39]. Given that the inhibition of plant IPCS can trigger SA-dependent HR-like lesions, such as those observed in response to AgN23 CME, we decided to study the implication of galbonolides in *Arabidopsis* responses to AgN23 [27]. We constructed AgN23 knock out mutants in the polyketide synthase of the galbonolides BGCs by disrupting the *gbnB* gene (AS97_41300) (Fig. 4A). Galbonolides detection was fully abolished in the CME of galbonolides knock-out mutants (Fig. 4B). This finding confirmed the function of the predicted galbonolide gene cluster in the synthesis of all the galbonolides detected (galbonolides A, B, E, and G).

To investigate the effect of AgN23 and galbonolide mutants CMEs on the IPCS activity, we prepared a microsomal fraction from a *Saccharomyces cerevisiae* strain producing recombinant *Arabidopsis* IPCS2 (AT2G37940) [30], and IPCS enzymatic activity was tracked by HPLC-Fluorescence method with the fluorescent substrate NBD-C6-ceramide [40]. Data were fitted to the Michaelis–Menten equation, the apparent Km and Vmax were estimated to be 7.57 μM and 0.01 mol/min/mg of protein, respectively [30] (See online supplementary material for a colour version of Supplementary Fig. S4).

We then tested the enzymatic activity in presence of AgN23 and mutant CMEs in the concentration range of 100–300 μg/ml. We observed that the AgN23 CME displayed a drastic inhibition of the enzymatic activity at concentrations > 200 μg/ml dilution (Fig. 4C) whereas no such inhibition was observed in the CME of two selected AgN23 galbonolides knock-out mutants (Δ*gbnB-2* and Δ*gbnB-4*). Taken together, these data revealed that galbonolides secretion by AgN23 is the driving factor in the inhibition of *Arabidopsis* IPCS2. Galbonolides were originally described as antifungal metabolites, the antifungal activity of the mutant was analysed against the filamentous fungus *Botrytis cinerea* [41, 42]. As expected, the loss of galbonolides in knock out mutants resulted in a reduced antifungal activity of the CME (See online supplementary material for a colour version of Supplementary Fig. S5 and Table 2).

**Table 2:** 50% inhibitory concentration (IC_50_) of AgN23 WT and KO mutants (Δ*gbnB*) CME against *Botrytis cinerea.* Table shows mean ± SD calculated from six biological replicates (n = 6).

Strain	IC_50_ (μg/ml)
AgN23 WT	33.28 ± 0.20
*ΔgbnB-1*	48.06 ± 1.68
Δ*gbnB-2*	47.27 ± 1.29
Δ*gbnB-3*	46.13 ± 1.29
Δ*gbnB-4*	54.42 ± 3.7

### Galbonolides are major contributors of the AgN23 eliciting activity and play a crucial role in rhizosphere colonization by AgN23

**Figure 5:**
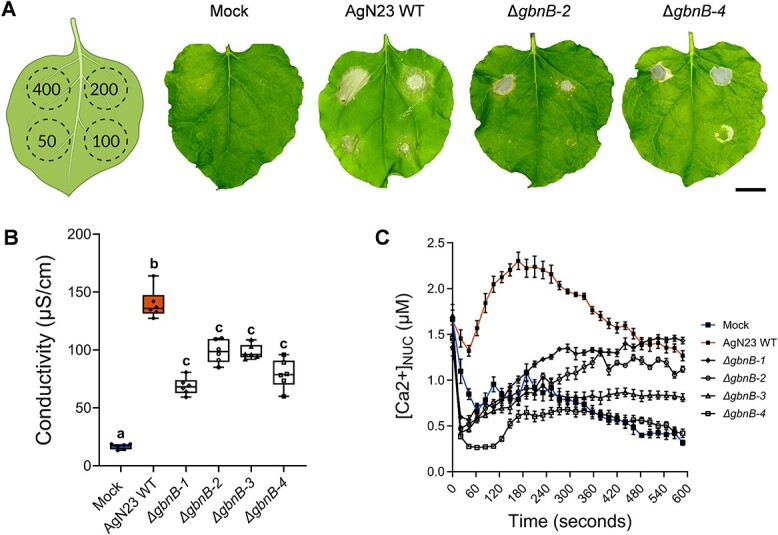
**The culture media extract of AgN23 triggers a galbonolides-dependent hypersensitive response. A.** Typical photographs of necrotic symptoms in *Nicotiana benthamiana* leaves 48 h after infiltration with CME of AgN23 WT or KO mutants (Δ*gbnB-2* and Δ*gbnB-4*) at 50, 100, 200 and 400 μg/ml as indicated in the scheme (n = 6). Scale bar: 3 cm. **B.** Ion leakage measurements of *Arabidopsis* leaf disks infiltrated with CME of AgN23 WT or KO mutants (Δ*gbnB*) at 100 μg/ml. box plots were created with data from six independent assays involving 5 to 6 leaf disks (n = 6). The letters a–c indicate statistically significant differences according to one-way analysis of variance (ANOVA) and Tukey’s HSD test (honestly significantly different, α = 0.05). **C.** Kinetics of AgN23 or KO mutants CME-induced nuclear calcium influxes in *Arabidopsis seedlings expressing* nuclear-localized aequorin. CME at 100 μg/mL was added at time = 0 min. Graphs show the mean ± SD calculated from 10 independent assays involving 3 plants per treatment (n = 10).

In a previous study, we identified AgN23 as a *Streptomyces* strain producing strong elicitors of the hypersensitive reaction (HR) including localized necrosis and expression of defence markers such as *Pathogenesis Related 1* (*PR1*), *Phytoalexin Deficient 4* (*PAD4*)*,* and *Phytoalexin Deficient 3* (*PAD3*) [27]. Here, we investigated whether galbonolides may play important role in these responses to the bacterium. Agroinfiltration of *Nicotiana benthamiana* leaves with AgN23 CME induced cell death at 50 μg/ml concentration whereas no sign of necrosis could be observed at the same concentration with CMEs of the galbonolides mutants Δ*gbnB-2* and Δ*gbnB-4* (Fig. 5A). However, similar necrotic responses were observed when CME of the wild type and mutant strains were infiltrated at 200 μg/ml or higher concentrations, suggesting that other necrotic elicitors were produced by the mutants. To investigate the effect of AgN23 CME on the necrotic responses of *Arabidopsis*, we performed ion leakage assays from infiltrated leaf discs of *Arabidopsis* with the four independent mutants of AgN23 and further confirmed the reduction in necrotic responses triggered by AgN23 when galbonolides biosynthesis is abolished (Fig. 5B). As variation of nuclear calcium concentration is a typical signal associated with HR, we analysed the nuclear calcium concentration of *Arabidopsis* plants following treatment using a line expressing a nuclear apo-aequorin reporter gene. This reporter line was also selected based on a previous observation that a nuclear calcium signal controls the apoptotic cell death induced by d-*erythro*-sphinganine, a compound related to the sphingolipid pathway, in tobacco cells [43]. Luminescence quantification triggered by AgN23 CME in hydroponically grown *Arabidopsis* peaked at 4 minutes post treatment and this signature was abolished in the galbonolides mutants (Fig. 5C). Similarly, we analysed by live imaging *Arabidopsis* seedlings inoculated at the root tip with AgN23 CME and observed that this treatment resulted in a quick activation (<15 min) of nuclear calcium signalling in the root tip which then spread to the entire root plantlets (Supplementary Movie).

**Figure 6:**
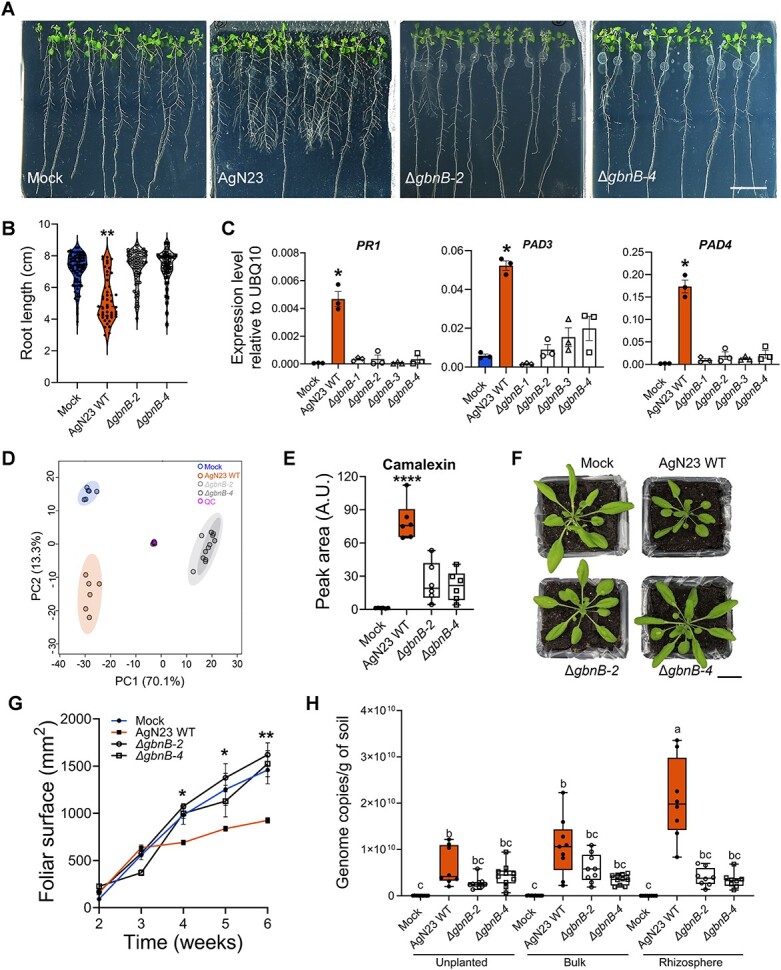
**Galbonolides play a crucial role in defense gene activation, camalexin biosynthesis and AgN23 persistence in the rhizosphere. A.** Observation of *Arabidopsis thaliana* Col-0 colonization by AgN23 WT or KO mutants at 10 days after inoculation with spores at the root apex. scale bar: 2 cm. **B.** Primary root length of plants colonized by AgN23 WT or KO mutants (Δ*gbnB*) at 10 days after inoculation. Statistical differences between the treatments were analysed using Mann–Whitney test and “^*^^*^^*^^*^” represents significant differences at *P* value <0.0001. **C.** Analysis of *PR1*, *PAD3*, and *PAD4* defense gene expression in 10-day old *Arabidopsis* seedlings at 6 hours after treatment with AgN23 CME. Graphs show the mean 2^-ΔCp^ relative to *UBQ10* and SD calculated from three biological replicates (n = 3), each involving five plants. Statistical comparisons were performed with t-test (“^*^” = *P* value <0.05). **D.** PCA score plot of UHPLC-HRMS data (n = 256 variables) from extracts of *A. thaliana* 10 days after inoculation with AgN23 WT or KO mutants (Δ*gbnB*) **E.** Average peak area of camalexin. Box plots were created from data from six independent assays (n = 6). The whiskers encompass the minimum and maximum values, and the midline shows the median. Statistical differences between the treatments were analysed using unpaired t-test and “^*^^*^^*^^*^” represents significant differences at *P* value <0.0001. **F.** Typical photographs of 6-week-old Col-0 rosettes grown in potting soil inoculated with AgN23 WT or KO mutants spores. Scale bar: 1.7 cm. **G.** Leaf area measurement. Graphs show the mean ± SD calculated from at least eight biological replicates (n = 8). **H.** AgN23 WT and Δ*gbnB-2* and Δ*gbnB-4)* mutants genome copy number in Col-0 rhizosphere 6 weeks after soil inoculation. Box plots were created from data from 8 plants per treatment (n = 8). The whiskers encompass the minimum and maximum values, and the midline shows the median. The letters a–c represent statistical differences between the treatments based on 2-way ANOVA followed by Tukey’s multiple comparisons test.

To investigate the impact of galbonolides production on root development, in vitro grown seedlings were inoculated with galbonolides mutants and no root growth inhibition was observed with the two mutants (Fig. 6A and B). Furthermore, the robust induction of expression in *PR1*, *PAD3,* and *PAD4* by AgN23 CME was compromised when using the four galbonolides KO mutants (Fig. 6C). Thus, our data demonstrate that galbonolides are required for the activation of immune gene expression in *Arabidopsis* seedlings in response to AgN23.

In view of our finding that *Arabidopsis* responds to AgN23 by strongly activating camalexin biosynthesis, we studied the effect of in vitro spore inoculation of Col-0 with AgN23 and the two galbonolide mutants Δ*gbnB-2* or Δ*gbnB-4*, by LC-HRMS metabolic fingerprinting (Supplementary Table S5). The PCA revealed a significant difference in the metabolome response to the two AgN23 mutants as compared to the wild type (Fig. 6D). The induction of camalexin detection was significantly lower in roots inoculated with galbonolides mutants as compared with the wild type bacterium (Fig. 6E).

Soil inoculation with galbonolides mutants did not resulted in the reduced growth of Col-0 rosette triggered by the wild-type bacteria (Fig. 6F and G). In addition, Col-0 rhizosphere colonization by galbonolides mutants was reduced by contrast with wild type AgN23 (Fig. 6H). Put together, these data clearly point to the crucial role played by galbonolides for the induction of plant responses as well as the ability of the bacterium to colonize the rhizosphere.

## Discussion

Understanding the chemical basis of the communication between plants and their associated microorganisms is essential to improve the function and composition of plant microbiota, specifically in the context of developing sustainable agriculture practices. Towards this effort, *Streptomyces* species could play a major role due to their ability to efficiently colonize the rhizospheric niche and to produce a wide array of specialized metabolites with various biological activity. However, mechanisms involved in the establishment and long-term maintenance of active microbial strains in the rhizosphere are largely unknown.

To gain insight into these mechanisms, we focussed on a *Streptomyces* strain, AgN23, initially isolated from the grape rhizosphere and that efficiently colonizes the rhizosphere of *A. thaliana*. Metabolic fingerprinting of the *Arabidopsis* response to AgN23 revealed that the response is mainly characterized by the production of camalexin, which is the primary *Arabidopsis* phytoalexin involved in resistance to fungal pathogens but also in the regulation of root microbiota composition and the recruitment of PGPRs [44–46]. The use of *pad3-1* camalexin deficient mutant of *Arabidopsis* demonstrated that the efficient colonization of the rhizosphere by AgN23 relies on the production of this compound. Although camalexin is an antimicrobial compound, the *pad3-1* mutants did not show any signs of over colonization by AgN23, suggesting that camalexin does not act as an inhibitor of AgN23 development but, on the reverse, favours colonization of the rhizosphere by AgN23. Albeit camalexin is produced in response to a number of bacterial and fungal phytopathogens, this does not mean it is biologically active against these microorganisms [47]. It was reported that concentrations up to 500 μg/ml are required to achieve membrane disruption in Gram negative bacteria, a range of concentration unlikely to be observed in or around *Arabidopsis* roots [48]. By contrast fungal colonizers of plant roots are sensitives to lower doses of camalexin [48]. Thus, the precise role of camalexin in supporting the development of AgN23 in the rhizosphere remains to be elucidated, but it can be hypothesized that camalexin can reduce the proliferation of susceptible fungi increasing available nutritional resources for AgN23. Recently, it has been shown that camalexin, and more generally tryptophan-derived metabolites, has been shown to be essential to prevent fungal dysbiosis in the *Arabidopsis* rhizosphere [49].

To understand the molecular mechanisms underlying the induction of camalexin biosynthesis by AgN23, we investigated the composition of the bacteria exometabolome using untargeted metabolomic tools. This analysis identified several specialized compounds with known antimicrobial activity and for some of them, a putative function in eliciting plant defences. Because galbonolides target the sphingolipid metabolism by inhibiting the IPCS in both plants and fungi, we decided to delve into their role in AgN23’s biological activities [33]. Sphingolipids are signalling molecules known to play a major role in plant defence [50], and the activation of camalexin biosynthesis [36]. Fungal toxins acting on this metabolism such as Fumonisin B1, an inhibitor of ceramide synthase produced by pathogenic *Fusarium* spp. may result in locally modifying the ceramide composition leading to induction of a hypersensitive response [51, 52]. To investigate the role of galbonolides in the induction of plant defences by AgN23 we produced galbonolide mutants through the disruption of a single BGC, confirming earliest reports indicating that all galbonolide variants are produced through a single BGC [53–56]. Using these mutants we performed a set of complementary experiments which pointed to the major requirement of galbonolides to trigger plant responses to AgN23 colonization.

The lack of enrichment of galbonolide mutants in the *Arabidopsis* rhizosphere shows that induction of plant defence by these compounds are beneficial for the AgN23 rhizospheric lifestyle. The connections between plant immune responses and stimulation of root microorganisms has been recently exemplified [57–60]. For example, the plant growth promoting rhizobacteria (PGPR) *Pseudomonas* sp. CH267 triggers the production of camalexin [44, 45]. Similarly, *Arabidopsis* root inoculation with the proteobacteria *Pseudomonas simiae* WCS417 results in the secretion of scopoletin, a coumarin that facilitates *P. simiae* root colonization while inhibiting the growth of fungal pathogens and diverse other bacterial taxa [61].

However, to our knowledge, the role of a specific microbial compound in eliciting plant defense responses for the benefit of the microganism has not yet been described and this result raises an interesting question about the generality of the role of galbonolides in the rhizospheric microbiota. Even though IPCS and the role of sphingolipid metabolism in immune responses are ubiquitous in plants, the distribution of the galbonolide in the *Streptomyces* genus, and more generally in actinomycetes, remains to be precised. In our previous study we showed that the galbonolide BGC is present in several species across the *S. violaceusniger* clade, to which AgN23 belongs, which includes several rhizospheric isolates [28]. In addition, the fact that galbonolides were initially found in *Streptomyces galbus* which does not belong to the *S. violacesuniger* clade, and in the distantly related actinomycete *Micromonospora* spp*.* suggests that the biosynthesis of galbonolides may be widespread across actinomycete representatives [53, 56, 62–64]. Further studies will aim to evaluate the impact of galbonolide production of microbiota functioning through the direct antifungal activity of these compounds and their impact on the production plant anti-fungal metabolites.

## Supplementary Material

Supplementary_Figures_Nicolle_et_al_S1-5_wrae112

supplementary_table_nicolle_et_al_proof_wrae112

Supplementary_Movie_wrae112

Supplementary_movie_legend_wrae112

Supplementary_methods_Nicolle_et_al_2024_wrae112

## Data availibility

Datasets generated or analysed during this study are included in this published article (and its Supplementary Table files). Raw data regarding RNAseq analysis and their complete analysis can be found on the NCBI Gene Expression Omnibus (GSE119986) [27]. The genome assembly of AgN23 is available on the NCBI “Nucleotide” repository (NZ_CP007153.1) with the chromosome sequence and the automatic annotation pipeline used to define gene models [28]. The LC-HRMS chromatograms of AgN23 CME and *Arabidopsis* are available on Zenodo repository (8421008).
