# Supplementary material for: Root-associated Streptomyces produce galbonolides to modulate plant immunity and promote rhizosphere colonization
Source: ISME J. 2024 Jun 19;18(1):wrae112. doi: 10.1093/ismejo/wrae112 (PMC11463028; doi:10.1093/ismejo/wrae112)
Supplement: Supplementary_Figures_Nicolle_et_al_S1-5_wrae112 [file supplementary_figures_nicolle_et_al_s1-5_wrae112.pdf]

Root-associated *Streptomyces* produce galbonolides to modulate plant immunity and promote rhizosphere colonization

Clément Nicolle[1], Damien Gayrard[1,2], Alba Noël[3], Marion Hortala[1], Aurélien Amiel[1,2], Sabine Grat[1], Aurélie Le Ru[4], Guillaume Marti[1,5,6], Jean-Luc Pernodet[3], Sylvie Lautru[3], Bernard Dumas[1*], Thomas Rey[1,2*]

AFFILIATIONS

1 Laboratoire de Recherche en Sciences Végétales, Université de Toulouse, CNRS, Université Toulouse III, Toulouse INP, 24 Chemin de Borde Rouge, Auzeville, 31320, Auzeville-Tolosane, France

2 DE SANGOSSE, Bonnel, 47480, Pont-Du-Casse, France

3 Université Paris-Saclay, CEA, CNRS, Institute for Integrative Biology of the Cell (I2BC), 91198, Gif-sur-Yvette, France

4 Plateforme d'Imagerie FRAIB-TRI, Université de Toulouse, CNRS, Auzeville-Tolosane 31320, France

5 Metatoul-AgromiX Platform, LRSV, Université de Toulouse, CNRS, UPS, Toulouse INP, Toulouse, France,

6 MetaboHUB-MetaToul, National Infrastructure of Metabolomics and Fluxomics, Toulouse, France

***Contributed equally**

CORRESPONDING AUTHOR'S EMAIL

bernard.dumas@univ-tlse3.fr

reyt@desangosse.com

LEAD CONTACT

Further information and requests for resources and reagents should be directed to the lead contact Thomas Rey. reyt@desangosse.com

SHORT TITLE

Galbonolides from rhizosphere *Streptomyces*

KEY WORDS

Rhizosphere, Galbonolides, *Streptomyces*, *Arabidopsis*, Camalexin

A

TAIR10 Id	Name	Annotation	AgN23 1 hpi	AgN23 6 hpi
At5g60890	ATMYB34	MYB transcription Factor	-1.689	-1.77
At1g18570	MYB51	MYB transcription Factor	4.54	5.32
At1g74080	ATMYB122	MYB transcription Factor	12.378	14.321
At4g39950	CYP79B2	Cytochrome P450	-1.093	5.684
At4g31500	CYP83B1	Cytochrome P450	-1.075	2.754
At2g30750	CYP71A12	Cytochrome P450	18.592	55.686
At2g30770	CYP71A13	Cytochrome P450	-1.294	153.13
At3g26830	PAD3	Cytochrome P450	28.805	29.805

B

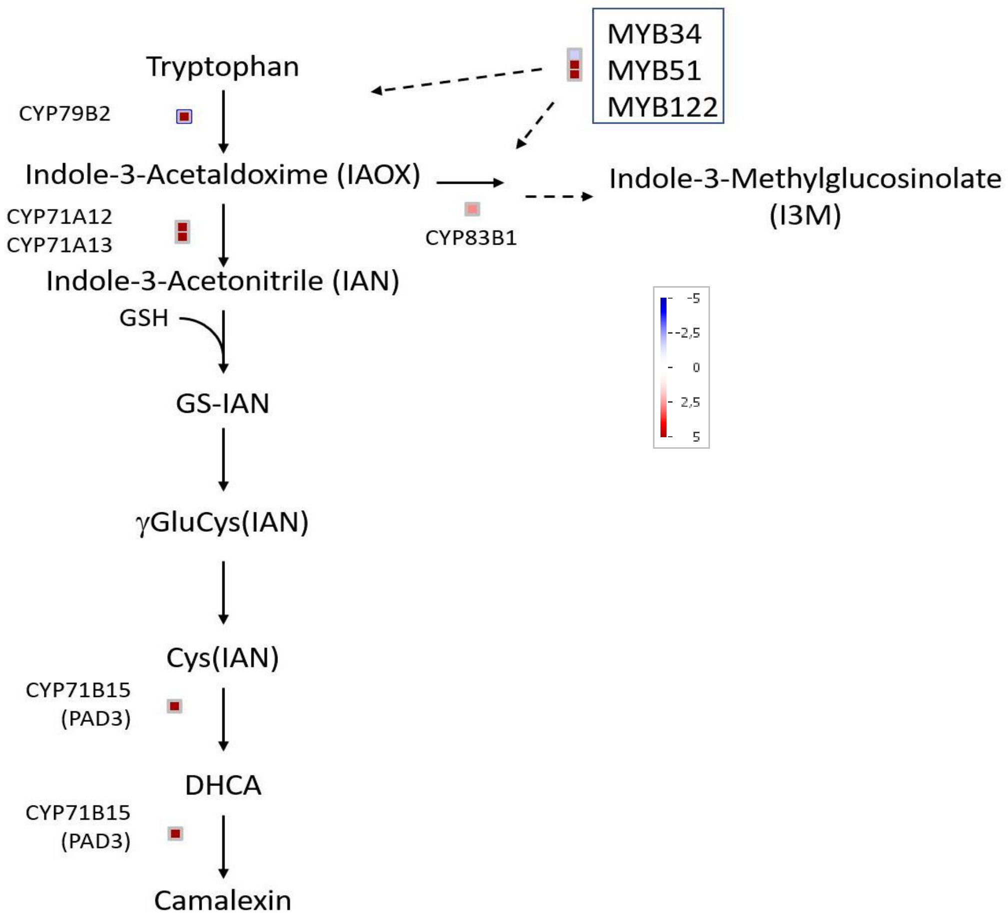

Supplementary Figure S1: Expression of genes involved in the biosynthesis of indolic compounds following treatment of *Arabidopsis* seedlings with AgN23 CME. Transcriptomic data from Vergnes et al., 2020 were mined to extract expression of genes falling in the category of indolic biosynthesis. **A.** Fold induction or repression expressed in Log2 of genes involved in gene regulation (MYB transcription factors) or camalexin biosynthesis at 1 hour post inoculation (hpi) and 6 hpi. **B.** Mapman display of gene regulation at 6 hpi from the biosynthesis pathway of camalexin and I3M (adapted from Ferigmann et al., MPMI Vol. 34, No. 5, 2021, pp. 560–570).

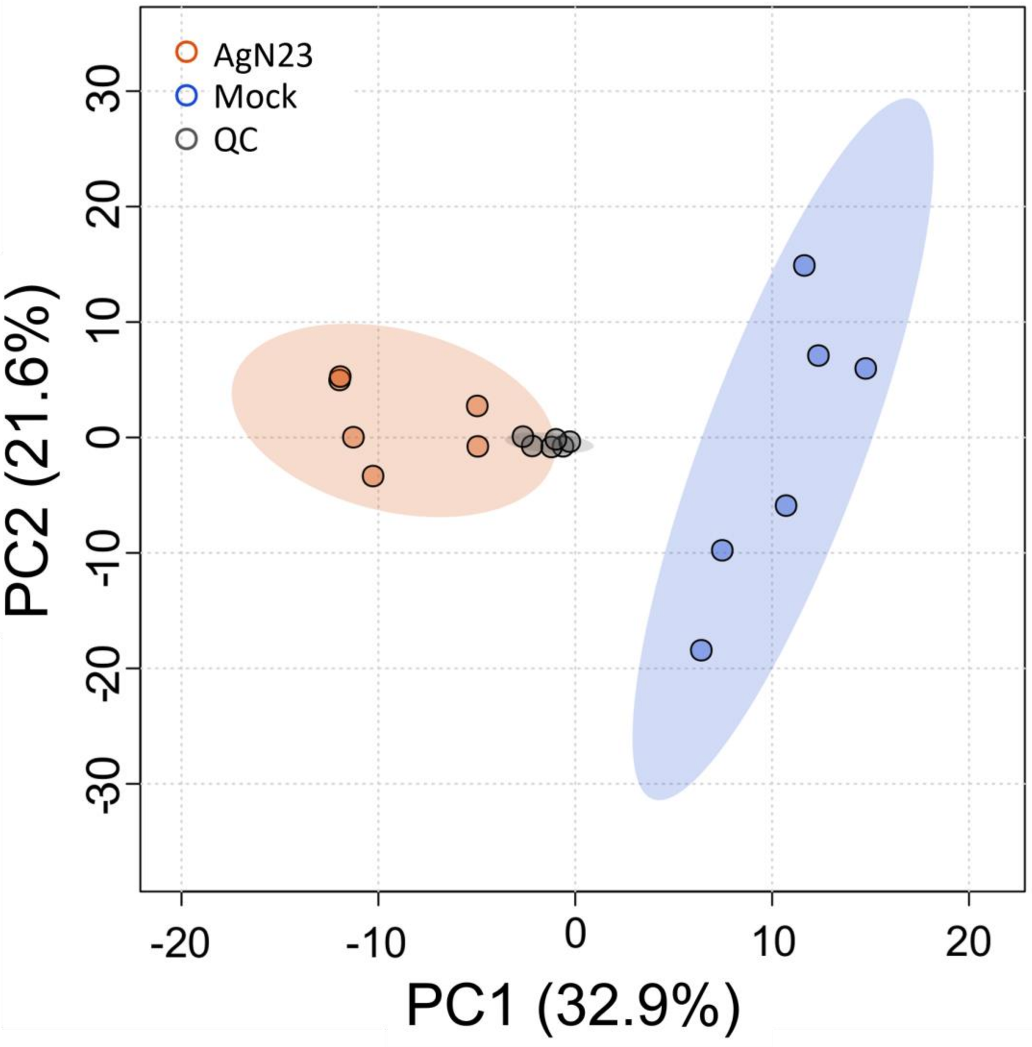

Supplementary Figure S2: PCA score plot of UHPLC-HRMS data (n = 511 variables) from extracts of *Arabidopsis thaliana* 10 days after inoculation or not with AgN23 spores. QC: quality control

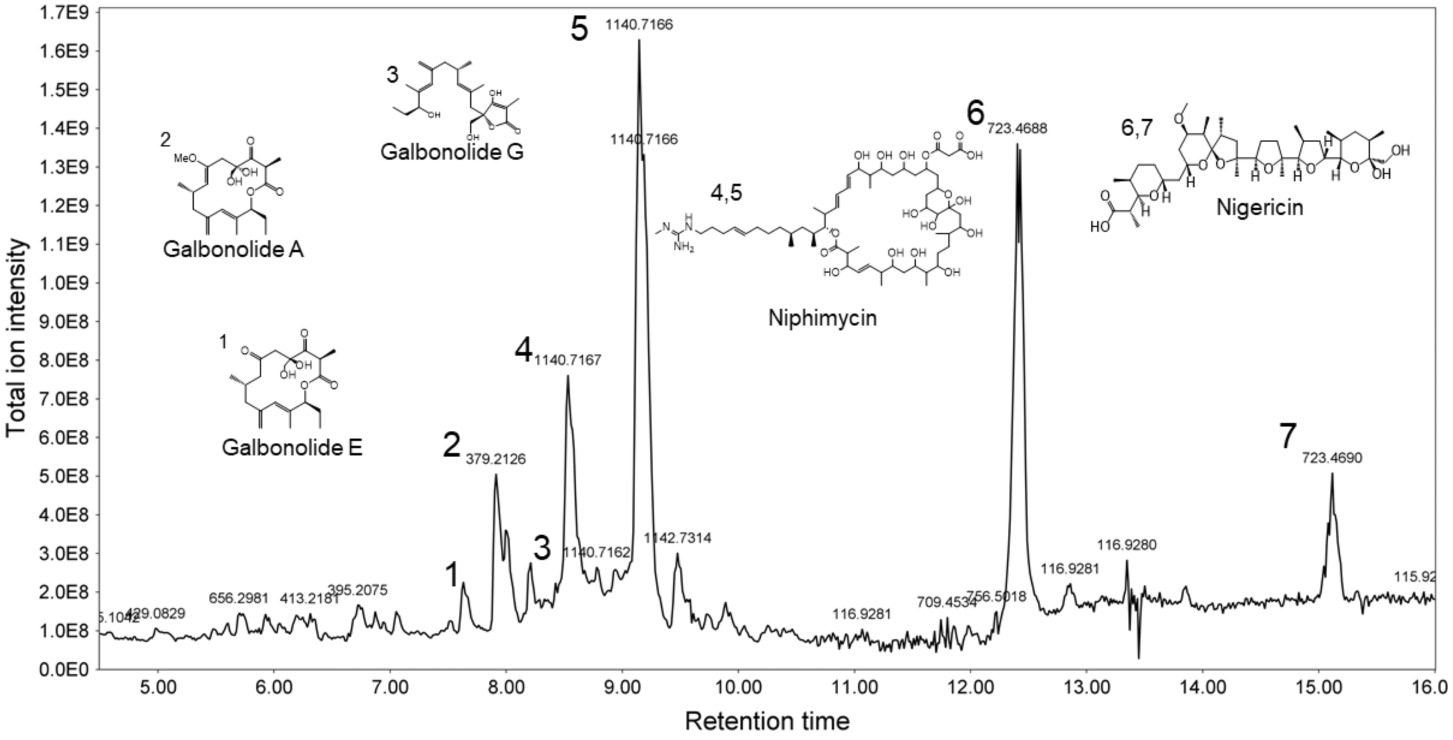

Supplementary Figure S3: UHPLC-HRMS chromatogram of AgN23 CME expressed in Total Ion Intensity. Peaks with the highest intensities were annotated with putative structures based on HRMS and MS/MS spectra.

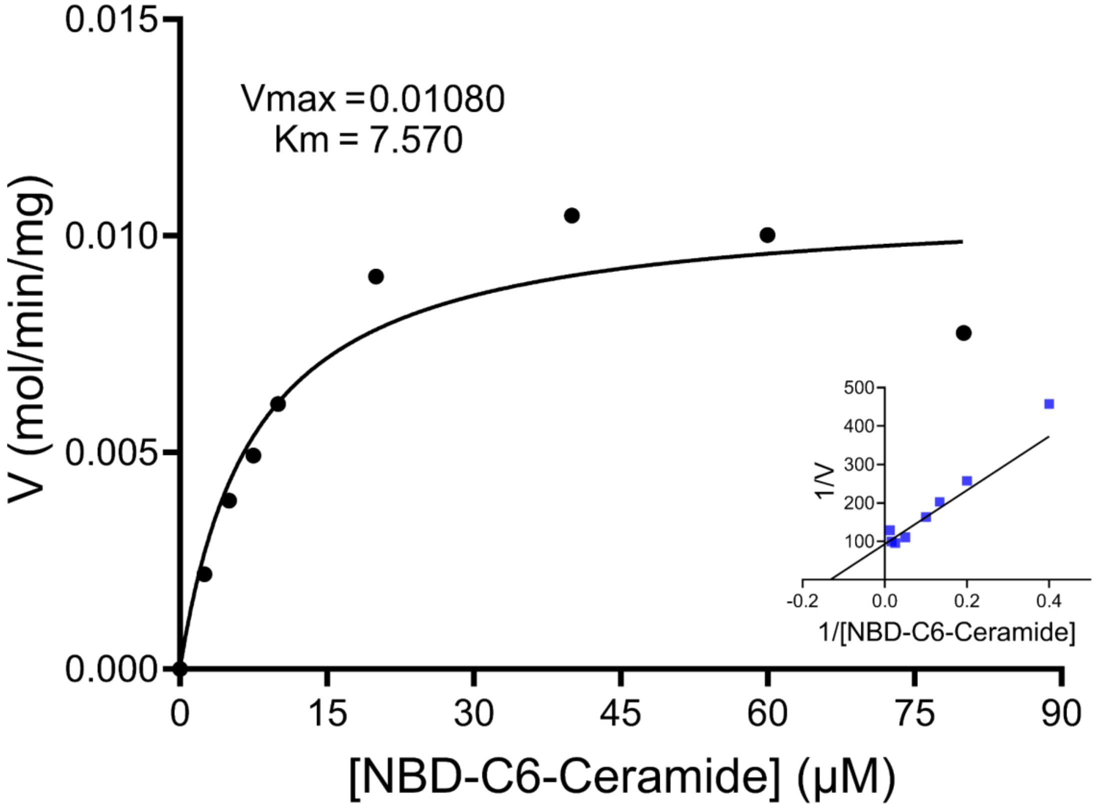

Supplementary Figure S4: Michaelis-Menten and Lineweaver Burk Vmax, and Km estimations from enzyme assays of the Inositol Phosphoceramide synthase from Arabidopsis thaliana (AtIPCS2). 0.1 mg/mL of total microsomal membranes were used to study the conversion of and the NBD-C6-Ceramide to NBD-C6-IPC. The fluorescence values of the assays were converted to concentrations based on the line of best fit from the standard curve of NBD-C6-Ceramide (3–500 µM).

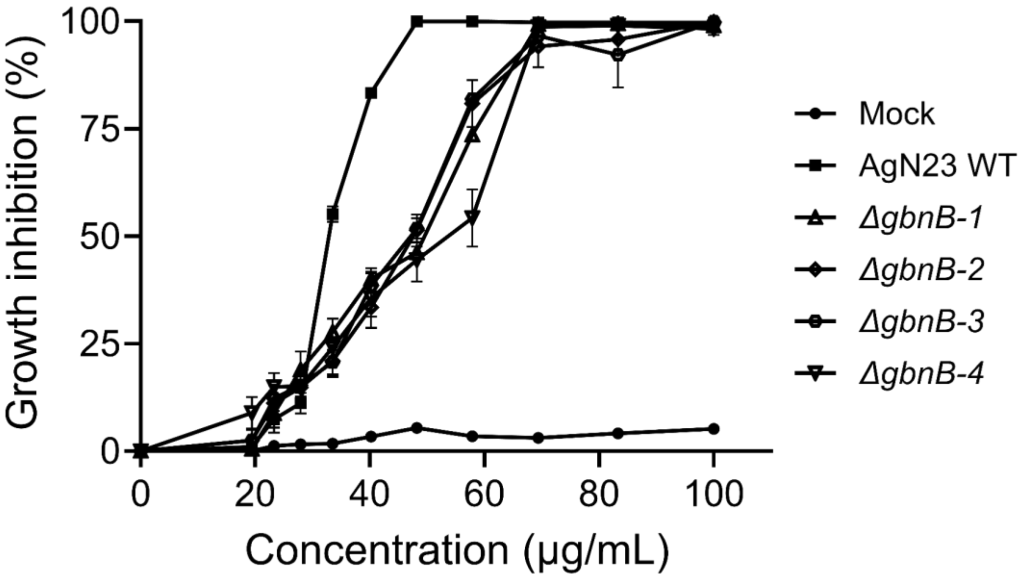

Supplementary Figure S5: Growth inhibition of Botrytis cinerea following treatment with CME of AgN23 WT and Δ*gbnB* mutants. Graphs show the mean ± SD calculated from six biological replicates (n = 6).
