# Supplementary figures and images for: Root-associated Streptomyces produce galbonolides to modulate plant immunity and promote rhizosphere colonization
Source: ISME J. 2024 Jun 19;18(1):wrae112. doi: 10.1093/ismejo/wrae112 (PMC11463028; doi:10.1093/ismejo/wrae112)

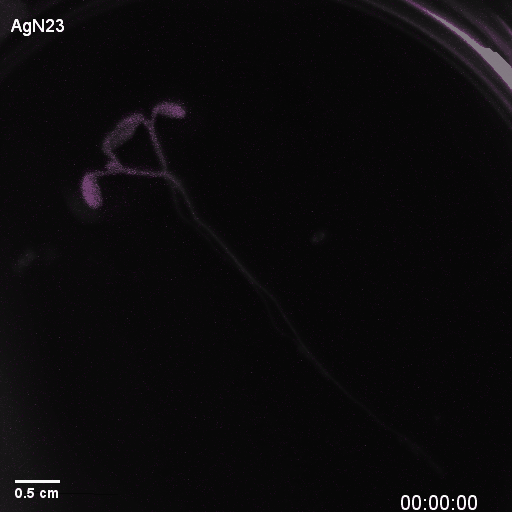

Supplement: Supplementary_Movie_wrae112 [file supplementary_movie_wrae112.gif]
