# Supplementary material for: Root-associated Streptomyces produce galbonolides to modulate plant immunity and promote rhizosphere colonization
Source: ISME J. 2024 Jun 19;18(1):wrae112. doi: 10.1093/ismejo/wrae112 (PMC11463028; doi:10.1093/ismejo/wrae112)
Supplement: Supplementary_movie_legend_wrae112 [file supplementary_movie_legend_wrae112.docx]

**Supplementary movie:** EM-CCD observations of calcium waves in the nuclei of an aequorin-expressing *Arabidopsis* seedling. Plants were inoculated at the root apex (white asterisk) with CME of AgN23 WT at 100 µg/mL (n = 3). White arrows show luminescent nuclei. Scale bar: 2 cm.
